# The Roles of Health Services on the Association between Medicare Annual Wellness Visits and Early Dementia Diagnoses

**DOI:** 10.1093/geroni/igaf122.2860

**Published:** 2025-12-31

**Authors:** Mukaila Raji, Huey-Ming Tzeng, Yong Shan, Jeong Jang, Peter Cram, Yong-Fang Kuo

**Affiliations:** University of Texas Medical Branch at Galveston, Galveston, Texas, United States; School of Nursing, University of Texas Medical Branch, Galveston, Galveston, Texas, United States; UTMB, Galvesto, Texas, United States; UTMB Health, The University of Texas Medical Branch, Galveston, Texas, United States; University of Maryland, Baltimore County, Baltimore, Maryland, United States; University of Texas Medical Branch, Galveston, Galveston, Texas, United States

## Abstract

Early recognition of cognitive impairment (CI) and timely diagnoses of mild CI (MCI) and Alzheimer’s Disease and Related Dementia (ADRD) are key to optimal dementia care. Currently there are no data describing the rates of guideline-informed health services (specific blood tests – vitamin B12, thyroid stimulating hormone; specialist referrals – neurology, psychiatry; and brain images – CT, MRI) conducted after Medicare Annual Wellness Visits (AWVs) in evaluation processes leading to MCI/ADRD diagnosis. Using propensity study cohort of Texas fee-for-service Medicare enrollees, (n = 66,433 at each group), we followed health services utilization and MCI/ADRD diagnosis within one-year of the first AWV or the index date for the non-AWV group in 2018. Both groups had similar proportion of patients who received specific blood tests, specialist referrals, and brain images in the 12 months before AWV/index date. Among those with no previous specialist visits, 4.1% in the AWV group and 3.5% in the non-AWV group got specialist referrals in the follow-up period. Among those with no previous brain images, 12.4% in AWV group and 11.7% in non-AWV group got brain images in the follow-up period. AWV receipt was associated with 41% increase in MCI diagnosis—with about 20% of the increase accounted for by post-AWV specialist referrals, lab tests, and brain images. AWV receipt was also associated with an 8% increase in ADRD diagnosis—with about 75% of the increase accounted for by the three guideline-informed health services. This study suggests that receipt of Medicare AWV has larger direct effect on the identification of early CI.

